# Increase in West Nile Neuroinvasive Disease after Hurricane Katrina

**DOI:** 10.3201/eid1405.071066

**Published:** 2008-05

**Authors:** Kevin A. Caillouët, Sarah R. Michaels, Xu Xiong, Ivo Foppa, Dawn M. Wesson

**Affiliations:** * Tulane University School of Public Health and Tropical Medicine, New Orleans, Louisiana, USA

**Keywords:** Natural disasters, West Nile virus, Hurricane Katrina, arbovirus, encephalitis, neuroinvasive disease, dispatch

## Abstract

After Hurricane Katrina, the number of reported cases of West Nile neuroinvasive disease (WNND) sharply increased in the hurricane-affected regions of Louisiana and Mississippi. In 2006, a >2-fold increase in WNND incidence was observed in the hurricane-affected areas than in previous years.

Hurricane Katrina devastated portions of Louisiana and Mississippi on August 29, 2005. Previous reports of West Nile neuroinvasive disease (WNND) in this area after this hurricane did not examine any statewide increases in 2005 (*1*). However, this report did not show potential regional increases of WNND in areas that experienced substantial hurricane damage. Because West Nile virus (WNV) is now endemic in areas of the United States that are at risk for hurricanes, understanding effects of such events on WNV epidemiology is important for directing appropriate public health responses. The objective of this study was to determine whether cases of WNND increased regionally after Hurricane Katrina.

## The Study

We used WNV human case data for Louisiana and Mississippi from the Centers for Disease Control and Prevention (CDC) (*2*); cases of meningitis, encephalitis, or meningoencephalitis reported to CDC were considered WNND cases. Cases are listed by date of onset of first symptoms and corresponding CDC week, and parish or county of residence at the estimated time of infection.

**Figure 1:**
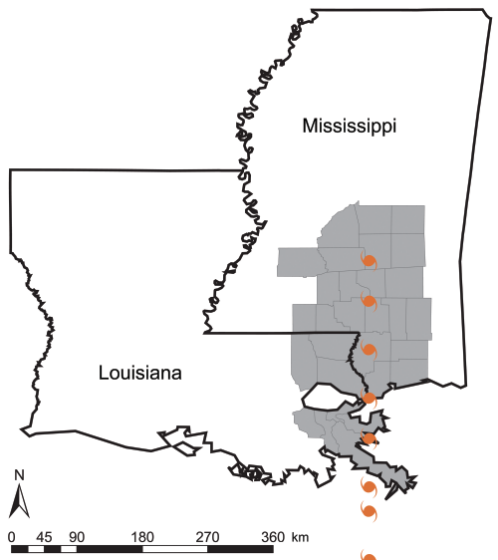
Hurricane Katrina track and hurricane-affected Louisiana parishes and Mississippi counties. Affected parishes and counties (gray) were defined as those in which >50% of the total area was <50 miles of the hurricane track coordinates.

Affected parishes or counties were defined as those in which >50% of the total area was within 50 miles of the hurricane track coordinates (*3*) (ArcView 8.0; Environmental Systems Research Institute, Redlands, CA, USA). Eight of 64 parishes in Louisiana and 21 of 82 counties in Mississippi fit our definition of hurricane affected (Figure 1). Counties within the storm’s track after its winds had diminished to <75 miles per hour were considered not affected.

We compared the number of WNND cases during the 3-week period before the storm with the number of cases in the 3-week period immediately after Hurricane Katrina to determine whether the number of WNND cases changed immediately after the storm in Louisiana and Mississippi. Because the hurricane-affected region experienced extensive migration of its residents and no valid population estimates exist for this period, the number of WNND cases reported was used. Landfall of Hurricane Katrina occurred at the beginning of CDC week 35, and news reports estimated that the final evacuation of persons from the New Orleans area occurred the following Sunday, September 4 (*4*), the beginning of CDC week 36. Because WNV infection has a 3–14-day incubation period (*5*), persons with storm-related exposures could have contracted WNV and become symptomatic as early as CDC week 35 or as late as the end of week 37. We considered WNND cases in which the reported onset of symptom dated from CDC weeks 35–37 as potentially influenced by the hurricane.

In Louisiana, no cases of WNND were reported in the 3 weeks before Hurricane Katrina (CDC weeks 32–34) in the 8-parish region affected by the storm. In the 3 weeks after the storm (CDC weeks 35–37), 11 WNND cases were reported in the affected region (Table 1). This increase in WNND cases in the hurricane-affected region was not observed during the same periods in 2002, 2003, 2004, or 2006. No increase was noted after the hurricane in unaffected parishes during the same periods.

**Table 1:** West Nile neuroinvasive disease (WNND) before and after Hurricane Katrina for the 3 years before the storm (2002–2004), the year of the storm (2005), and the year after the storm (2006) in Louisiana parishes and Mississippi counties*

State	Affected areas						Unaffected areas				
	CDC weeks 32–34†			CDC weeks 35–37‡			CDC weeks 32–34†			CDC weeks 35–37‡	
	WNND cases	95% CI		WNND cases	95% CI		WNND cases	95% CI		WNND cases	95% CI
Louisiana											
2002	22	14.6–33.3		8	4.1–15.8		37	26.9–51.0		20	13.0–30.9
2003	2	0.6–7.2		3	1.09–8.8		18	11.4–28.5		16	9.9–26.0
2004	1	0.2–5.6		1	0.2–5.6		23	15.4–34.5		11	6.2–19.7
2005	0	0–3.0§		11	6.2–19.7§		28	19.4–40.5		12	6.9–21.0
2006	11	6.2–19.7		8	4.1–15.8		13	7.7–22.2		16	9.9–26.0
Mississippi											
2002	13	7.7–22.2		12	6.9–21.0		38	27.7–52.2		21	13.8–32.1
2003	3	1.1–8.8		3	1.1–8.8		5	2.2–11.7		4	1.6–10.2
2004	1	0.2–5.6		2	0.6–7.2		12	6.9–21.0		4	1.6–10.2
2005	0	0–3.0§		10	5.5–18.4§		8	4.1–15.8		10	5.5–18.4
2006	12	6.9–21.0		9	4.8–17.1		14	8.4–23.5		9	4.8–17.1

*CDC, Centers for Disease Control and Prevention; CI, confidence interval (Poisson). †3 weeks before landfall of hurricane. ‡3 weeks after landfall of hurricane. §p<0.05.

A similar pattern was observed in Mississippi. In the 3 weeks after landfall, the affected region showed an increase from 0 to 10 WNND cases; the unaffected region of Mississippi showed only a minor increase in cases during the same periods (8 cases before and 10 cases after the storm).

To assess potential long-term effects of Hurricane Katrina on WNND incidence, we compared incidence rates of WNND for both states during 2006 with rates during the 4 years preceding the storm (2002–2005). Because the hurricane-affected region experienced population displacement, we used special population estimates from the US Census Bureau for rate estimations for 2006 (*6*). For unaffected parishes or counties that did not have an updated census estimate, we used the Census 2000 population estimate (*7*). Louisiana had population reductions of 398,853 persons (–28%) in hurricane-affected parishes and 17,521 persons (<–1%) in unaffected parishes. Mississippi had population reductions of 21,708 persons (–3%) in affected counties and 34,545 persons (–2%) in unaffected counties.

**Figure 2:**
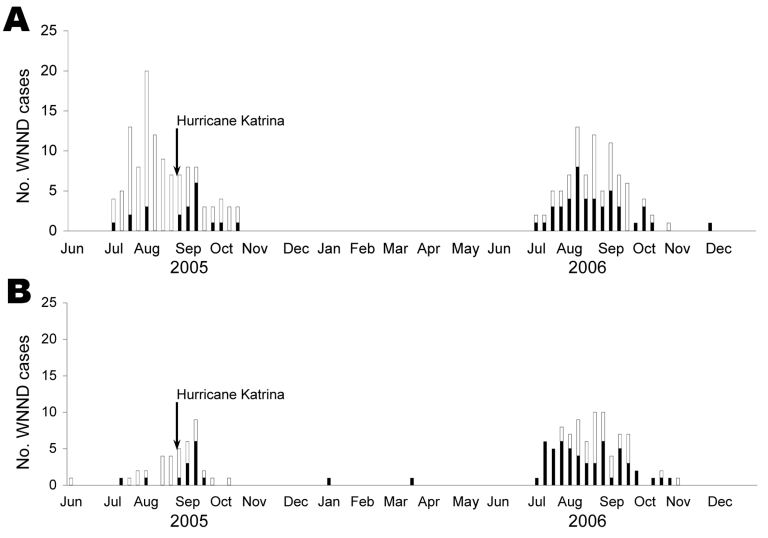
Cases of West Nile neuroinvasive disease (WNND) in Louisiana (A) and Mississippi (B), 2005–2006. Hurricane Katrina made landfall on August 29, 2005 (Centers for Disease Control and Prevention [CDC] week 35). An increase in WNND cases is noted in the hurricane-affected parishes and counties (black columns) during the 3 weeks after the storm (CDC weeks 35–37). Cases of WNND increased throughout the 2006 season in hurricane-affected parishes. Cases of WNND from unaffected parishes and counties are shown in white columns.

Despite losses in population, the affected parishes of Louisiana had an increase in the number of WNND cases from an average annual number of 30 cases in 2002–2005 to 45 cases in 2006. In the affected counties of Mississippi, WNND cases increased from an annual number of 23 cases in 2002–2005 to 55 cases in 2006. Incidence rate ratios and 95% confidence intervals were calculated for each state and region (affected and unaffected) (Table 2). Incidence rate ratios for 2006 were >2-fold higher in the hurricane-affected regions of both states than the mean historic incidence rates (2002–2005). Unaffected areas of both states showed decreased (Louisiana) and stable (Mississippi) WNND incidences compared with historical incidence. Figure 2 shows epidemic curves of 2005–2006 cases by week in affected and unaffected areas for both states.

**Table 2:** Incidence rate ratios of West Nile neuroinvasive disease (WNND) in 2002–2005 and 2006 in Louisiana parishes and Mississippi counties*

State, area	West Nile neuroinvasive disease incidence rate†						Incidence rate ratio (95% CI)
	2002‡	2003‡	2004‡	2005‡	2002–2005§	2006¶	
Louisiana							
Affected	5.6	1.3	0.2	1.4	2.1	4.4	2.09 (1.48–2.94)
Unaffected	4.1	2.7	2.7	3.2	3.2	1.5	0.47 (0.35–0.64)
Mississippi							
Affected	6.1	2.1	0.8	1.5	2.6	6.5	2.45 (1.77–3.47)
Unaffected	5.5	1.7	1.2	1.3	2.4	1.7	0.71 (0.55–1.03)

*WNND incidence rates increased 2-fold in the hurricane-affected regions of both states. The unaffected regions showed a decrease in WNND incidence rates (Louisiana) and no change in incidence (Mississippi). CI, confidence interval. †No. cases/100,000. ‡Population estimate based on 2000 US Census (*8*). §Cumulative WNND incidence = (no. WNND cases 2002 + 2003 + 2004 + 2005) / cumulative population (Census 2000 [*8*] × 100,000). ¶Population estimate based on 2006 US Census estimate (*7*).

## Conclusions

Our evidence demonstrates that areas directly affected by Hurricane Katrina experienced increases in WNND cases after the storm compared with before the storm. Analyses of the immediate period after the storm indicate that the observed increase was unique both in time and to the affected region. WNND incidence in 2006 equaled or exceeded the incidence rates in both states during the 2002 epidemic. Because WNND complications are seen in ≈1% of WNV infections (*5*), a small increase in WNND cases represents a much larger increase in WNV human transmission.

Because our study is based on surveillance data, confounding factors that may bias our analysis should be considered. Although Lehman et al. (*1*) indicated that case reporting lagged in Louisiana after Hurricane Katrina, no evidence was provided to suggest that year-end case totals were affected. To account for potential interstate reporting differences, we have conducted separate analyses for each state. Creation of 3-week periods on the basis of the day (August 29, 2005) and week (CDC week 35) that the storm made landfall may also introduce bias. Some cases with reported onset dates in the 2 weeks after the storm may have resulted from transmission events before August 29th. However, storm-related exposure to mosquitoes began before the storm’s landfall, when in preparation for the approaching storm, residents boarded windows and cleared yards. Despite these potential confounding factors, we believe the magnitude of the increase in WNND cases occurring immediately after Hurricane Katrina and the increases in WNND incidence in 2006, within the hurricane-affected region, is substantial enough to warrant further examination.

The immediate increase in cases may be attributed to increased human exposure to mosquitoes. Tens of thousands of persons in the hurricane-affected region were living in damaged housing or were waiting outside for days to be evacuated. The sudden decrease in WNND cases in the hurricane-affected areas 3 weeks after landfall could be attributed to reduced human exposure caused by eventual evacuation and aerial application of insecticides (*8*). The increase in WNND incidence in 2006 might also be due to increased human-mosquito exposure as a result of mosquito larval habitat creation (root ball voids from fallen trees, and flooded abandoned swimming pools), continued substandard living conditions, and increased outdoor reconstruction activities.

Hurricane Katrina was the first major tropical cyclone to make landfall in a large metropolitan area since the 1999 introduction of WNV into the United States. The scale of hurricane damage, especially to residences, may have contributed to increases in WNND. We recommend a region-specific, short- and long-term analysis of arboviral disease to accurately assess the public health effect of natural disasters. Prepositioning mosquito control assets and continuing to provide enhanced emergency assistance for surveillance and control could aid in inhibition of mosquito-transmitted diseases during the immediate period after a hurricane and throughout an extended recovery period.
